# A Method for Decomposition of the Basic Reaction of Biological Macromolecules into Exponential Components

**DOI:** 10.1186/s11671-016-1758-1

**Published:** 2016-12-07

**Authors:** Yu. M. Barabash, A. K. Lyamets

**Affiliations:** Institute of Physics, National Academy of Science of Ukraine, 46, Nauky Ave, Kyiv, 03039 Ukraine

**Keywords:** Multidimensional optimization, Decomposition of the curve, Exponential function, Electron-conformational states of biological macromolecules, Multi-component structure, Reaction center, Purple bacteria, Electron transport, Fluorescence of chlorophyll, Configuration variability

## Abstract

The structural and dynamical properties of biological macromolecules under non-equilibrium conditions determine the kinetics of their basic reaction to external stimuli. This kinetics is multiexponential in nature. This is due to the operation of various subsystems in the structure of macromolecules, as well as the effect of the basic reaction on the structure of macromolecules. The situation can be interpreted as a manifestation of the stationary states of macromolecules, which are represented by monoexponential components of the basic reaction (Monod-Wyman-Changeux model) Monod et al. (J Mol Cell Biol 12:88–118, 1965). The representation of multiexponential kinetics of the basic reaction in the form of a sum of exponential functions $\left(A(t)={\displaystyle {\sum}_{i=1}^n{a}_i{e}^{-{k}_it}}\right)$ is a multidimensional optimization problem. To solve this problem, a gradient method of optimization with software determination of the amount of exponents and reasonable calculation time is developed. This method is used to analyze the kinetics of photoinduced electron transport in the reaction centers (RC) of purple bacteria and the fluorescence induction in the granum thylakoid membranes which share a common function of converting light energy.

## Background

It is known that the kinetics of electron transport in the RCs of purple bacteria [1–3] and the shape of the curve of chlorophyll fluorescence in the complex of photosystem II in thylakoid grana are multiexponential in nature [4]. As a rule, this is explained by a multilevel organizational structure of macromolecules and a manifestation of self-regulation effect on the structural and dynamic properties of biological macromolecules in non-equilibrium operating conditions. This effect is determined by certain hidden parameters among which structural variables that characterize the state of macromolecules can act. While these parameters are difficult to measure directly in experiment, they impact the kinetics of basic reaction in macromolecules along with control parameters (substrate concentration, light intensity, etc.). Due to changes in the structure of macromolecules, a feedback in the basic reaction occurs. This feedback links the recurring elementary acts of the basic reaction. The elementary acts lead to significant changes in the structure of macromolecules. They modify the very elementary reaction and provide adaptive functioning of the macromolecules. The effect of self-regulation can occur with different amounts of structural variables. These factors make it difficult to analyze the basic reaction, and the kinetics of this reaction assumes a multiexponential character. Therefore, there is a problem, using the experimentally measured kinetics of basic reaction, to identify the stationary states and self-consistent reorganizations of macromolecules, which are reflected by monoexponential components of the basic reaction.

## Methods

### Multidimensional Optimization Method

The representation of multiexponential kinetics curve for the basic reaction in the form of a sum of exponential terms $\left(A(t)={{\displaystyle {\sum}_{i=1}^n{a}_ie}}^{-{k}_it}\right)$ with restriction (*k*_*i*_ *>* 0, *a*_*i*_ > 0), is a multidimensional optimization problem with identification difficulties. There are various optimization algorithms (gradient, evolutionary, stochastic methods, etc.) which have different sorting strategies. The choice of algorithm is related to the ambiguity of the behavior of objective function, the existence of local extrema of objective function, and the *curse of dimensionality* (obtaining quality results within a reasonable time) [5, 6].

To analyze the kinetics of photoinduced charge transport in the RCs of purple bacteria and induce the fluorescence in the granum thylakoid membranes, a gradient optimization algorithm using the methods of linear algebra was developed. This algorithm allows to determine the number of exponents and their parameters which correspond to the global minimum of the objective function expansion (root-mean-square error). It also allows excluding the impact of initial values of parameters and step size of their changes on the results of calculation. The algorithm does not require searching the values of exponential weights (*a*_*i*_) during optimization that provides a reasonable time of calculations.

The algorithm includes two reciprocal steps. At the first step, the problem of determining the numerical representation (*a*_*i*_) of a given decomposition basis with the best approximation to the analyzed curve is solved. At the second step, an optimization of the parameters of decomposition basis (*n*, *k*_*i*_ *,* (*a*_*i*_)) is carried out. Let us briefly consider the first step. One can assume that the analyzed signal *x*(*t*) with a finite energy belongs to a metric space with the scalar product (*x*, *y*) = ∫*x*(*t*)*y*(*t*)dt. The signal *x*(*t*) is uniquely (best) represented as a linear combination [7]

1 $$ x(t)={\displaystyle {\sum}_{i=1}^n{\alpha}_i{\varphi}_i(t),} $$

where φ_i_(*t*) is a system of *n* linearly independent functions, and α_i_ form the desired numerical representation *x*(*t*). This basis does not need to be orthogonal. Let us multiply *x*(*t*) by φ_j_(*t*). Then the relation between the signal *x* and its numerical representation *a* in a matrix form is

2 $$ \left[\begin{array}{c}\hfill \left({\varphi}_1,{\varphi}_1\right)\left({\varphi}_2,{\varphi}_1\right)\dots \left({\varphi}_n,{\varphi}_1\right)\hfill \\ {}\hfill \left({\varphi}_1,{\varphi}_2\right)\left({\varphi}_2,{\varphi}_2\right)\dots \left({\varphi}_n,{\varphi}_2\right)\hfill \\ {}\hfill .\hfill \\ {}\hfill .\hfill \\ {}\hfill .\hfill \\ {}\hfill \left({\varphi}_1,{\varphi}_n\right)\left({\varphi}_2,{\varphi}_n\right)\dots \left({\varphi}_n,{\varphi}_n\right)\hfill \end{array}\right]\left[\begin{array}{c}\hfill {a}_1\hfill \\ {}\hfill {a}_2\hfill \\ {}\hfill .\hfill \\ {}\hfill .\hfill \\ {}\hfill .\hfill \\ {}\hfill {a}_n\hfill \end{array}\right]=\begin{array}{c}\hfill \left(x,{\varphi}_1\right)\hfill \\ {}\hfill \left(x,{\varphi}_2\right)\hfill \\ {}\hfill .\hfill \\ {}\hfill .\hfill \\ {}\hfill .\hfill \\ {}\hfill \left(x,n\right)\hfill \end{array},\kern0.5em \boldsymbol{Ga}=\boldsymbol{\beta},, $$

Let us introduce reciprocal basis functions θ_*i*_(*i* = 1, 2, 3, …, *n*) which are represented by linear combinations ${\upvarphi}_i\left({\uptheta}_j\right)={\displaystyle {\sum}_{k=1}^n{\varGamma}_{\mathrm{jk}}{\upvarphi}_k(t)},$. The scalar products of the bases equal $\left({\upvarphi}_i,{\uptheta}_j\right)={\updelta}_{\mathrm{ij}},\kern0.5em i.e{\displaystyle {\sum}_{\boldsymbol{k}=\mathbf{1}}^{\boldsymbol{n}}\boldsymbol{\Gamma} \boldsymbol{j}\boldsymbol{k}\left({\boldsymbol{\varphi}}_{\boldsymbol{i}},{\boldsymbol{\varphi}}_{\boldsymbol{k}}\right)}={\boldsymbol{\delta}}_{\boldsymbol{i}\boldsymbol{j}},\kern0.1em \boldsymbol{\varGamma} \ast \boldsymbol{G}=\boldsymbol{I}\to \boldsymbol{\varGamma} ={\left[{\boldsymbol{G}}^{-\mathbf{1}}\right]}^{*}$ in matrix form. Using a common basis, it is possible to rewrite (2) in the following form:

3 $$ {\boldsymbol{\alpha}}_{\boldsymbol{i}}=\left(\boldsymbol{x},{\boldsymbol{\theta}}_{\boldsymbol{i}}\right);\kern0.75em \boldsymbol{i}=\boldsymbol{1},\boldsymbol{2},\dots, \boldsymbol{n} $$

where α_i_ are the required values of exponential weights.

Therefore, at the first step the number (*n*) and discrete values of decrements (*k*_*i*_) of the basic exponential functions φ_*k*_ are specified, and a matrix of their scalar products *G* is formed. Then we calculate the inverse to *G* matrix Г and determine their reciprocal basis θ_*j*_. Using the expression (3), we find the values of the weights of exponential functions *α*_*i*_, which best fit the analyzed signal. According to the array of possible decrements, the decrements of exponential functions (*k*_*i*_) are discrete in nature. This leads to errors in the optimization, which correspond to the difference between the left and right side of the expression (1), i.e. between the analyzed signal and its approximation.

Now the first step is finished. At the second step we find the optimal parameters of the decomposition basis. We select one exponent with an initial value of decrement and, using the operations of the first step, go over the decrement values *k*_1_ and determine the optimal values of the parameters (*α*_1opt_, *k*_1ont_, *a*_1_ > 0) of the first exponent, which provides the minimum error (dispersion) of approximation. Then we add the second exponent and use the first step of optimization. At fixed value of the first exponent, *k*_*1opt*_, we examine *k*_2_ and determine the optimal values for two exponents (*a*_1opt_, *k*_1ont_, *a*_2opt_, *k*_2ont_). After that we go back to the first exponent and at fixed value of the second exponent, *k*_2opt_ go over *k*_1_ and determine new optimal values of both exponents. Then we return to the second exponent and at new value of *k*_1opt_ examine *k*_2_ and determine new values of both exponents. The procedure is repeated until the dispersion of approximation stops to change. Then we add the third exponent and determine *k*_3opt_ for it with fixed decrements of previous two exponents, *k*_1opt_ and *k*_*2*opt_. After that we repeat the above process of optimization for two exponents with fixed value of the third exponent *k*_3opt_. Then we adjust *k*_3opt_ for new values of *k*_1opt_ and *k*_*2*opt_. Next, we adjust *k*_1opt_ and *k*_*2*opt_ at fixed value of *k*_3opt_. The procedure is repeated until the dispersion of approximation stops to change. Then we add the fourth exponent, and so on. The optimization process ends when the next set of the components of decomposition at the next step will have greater dispersion than the previous set, and the latter will be used.

**Fig. 1:**
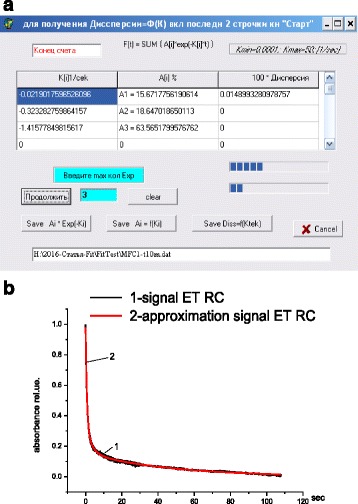
**a** Menu of the program for signal decomposition into sum of exponential functions. **b** Plots of experimental and approximation signals (both normalized)

Computer software for the above algorithm has been developed. The program menu is shown in Fig. 1a. The analyzed signal should be in text format in two columns, with the point as decimal mark. The first column includes time (*X* axis) in seconds, and the second column contains signal amplitude in arbitrary units. The signal amplitude should continuously reduce with time. To run the program, one has to enter in the menu the maximal number (<8) of decomposition exponents and click *Start/continue*. As a result, the directory with files appears.

If the original file has correct format, the decomposition of the initial signal into exponential components starts. After that the parameters of exponents will appear in a table. The decomposition results can be saved in two text files: (i) as an approximation signal for visual control, by means of Save Ai*Exp(*-Ki*) button, or (ii) as a table of exponential parameters, by clicking *Save Ai = f*(*Ki*). Figure 1b shows the normalized plot of the kinetics of absorption recovery of RC solution after switching off the excitation light (reference signal) and the approximation curve for the experimental signal. A good correlation between the experimental signal and its decomposition into three components (dispersion = 0.00015) is observed. The accuracy of decomposition is ±2.5%.

**Fig. 2:**
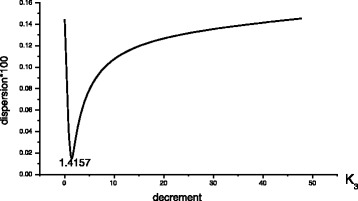
Dependence of objective function (dispersion) of decomposition on the decrement *k*_3_ at the final stage of optimization (after *k*_1_ and *k*_2_ were decrement)

Figure 2 shows a characteristic behavior of the objective function of approximation of electron transport kinetics in RC for different modes of photoexcitation. This character of the objective function allows the optimization without the risk of falling into a local minimum, and significantly reduces the computation time (to 5 min).

## Results and Discussion

### Problem Solving and Discussion

The developed software has been tested for an analysis of the kinetics of electron transport in various modes of RC photoexcitation and induction of chlorophyll fluorescence in four leaves of winter wheat.

The RCs were studied on wild-type isolated bacteria *Rhodobacter sphaeroides*. The chromatophores of bacteria were solubilized by detergent LDAO (lauryldimethylamine *N*-oxide), and RCs were separated from other membrane components by means of hydroxyapatite column chromatography. The isolated RCs were slurry suspended in a 0.01-M Na-P buffer, pH 7.2, with 0.05% LDAO [8]. The RC absorption kinetics was studied by means of exposure of RCs to light pulses of different durations and intensities. The results were satisfactorily approximated by 2 ÷ 4 exponential functions.

**Fig. 3:**
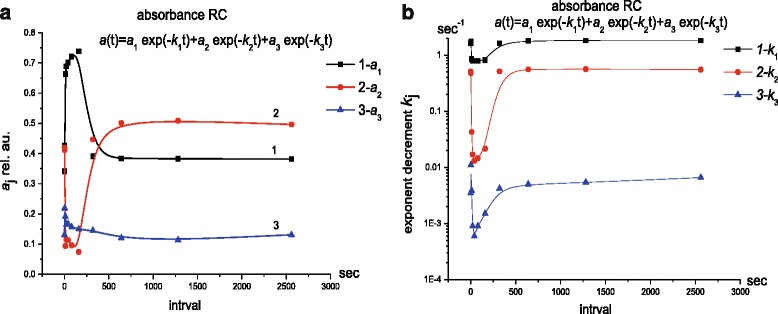
**a**, **b** The parameters of exponential components of kinetic curve of RC recovery *a*_*i*_ and *k*_*i*_ after the exposure to second light pulse as a function of the interval between two successive pulses of RC photoexcitation

Although the absorption kinetics corresponded to the kinetics of electron transport, the achievement of dark absorption values during RC relaxation after turning off the light did not guarantee the end of the structural changes in RCs. In this regard, the RC absorption kinetics was studied under photoexcitation by two successive light pulses with duration of 100 s and intensity of 7 mW/cm^2^; the interval between the pulses was varied from 0 to 2500 s (Fig. 3).

The present studies showed that at intervals between RC photoexcitation pulses smaller than 40 s the weight *a*_*1*_ of fast component of RC recovery increased, the weight *a*_*2*_ of intermediate component decreased, and the weight *a*_*3*_ of slow component slightly increased; the decrements *k*_*1*_,>*k*_*2*_,>*k*_*3*_ rapidly dropped, that indicated on the impact of previous RC history. At intervals *t*_*imp*_ **=** 40 ÷ 100 s a slow change of parameters was observed. At *t*_*imp*_ > 100 s the parameters of exponents reached their initial values. At intervals greater than 700 s, the parameters (*a*_*i*_, *k*_*i*_ ) of RC recovery after turning off the second pulse did not change, and the pulses of photoexcitation were independent of each other.

The efficiency of the developed algorithm was also tested in the study of fluorescence induction in the PSII chlorophyll complexes of four types of winter wheat of different ages. The shape of chlorophyll fluorescence induction curve in the leaves of plants depends on several processes that determine the energy distribution. A significant role is played by the energy exchange processes in the light-harvesting antenna complex, photochemical transformation, and electron outflow from the reaction centers. Blocking the electron transport from the reaction centers leads to a simplification of the shape of fluorescence curve. In this case the shape depends mainly on the energy transfer in antenna. Several subsystems (units) can be distinguished in the PSII structural organization [9, 10]: a unit of photoelectric converters that perform the photochemical reaction, an antenna unit, and block of small protein subunits. The antenna unit includes pigment-proteins of internal antennas, external minor antennas, and external LHCII antennas with trimers (S, M, L). The PSII complex has three dimers (C2–dimers of core-complexes) which include the reaction centers with internal antennas, as well as trimers (S, M) of the extenal LHCII antenna. The induction of fluorescence was measured in a classical mode in cut leaves adapted to darkness. The electron transport was blocked by DCMU (3-(3,4-dichlorophenyl)-1,1-dimethylurea) which was fed into the tissues together with the buffer mixture through the section in the bottom of the leaves.

**Fig. 4:**
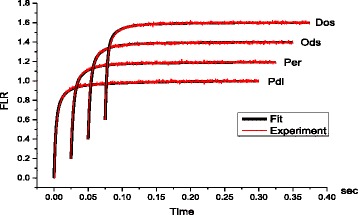
Experimental curves of fluorescence induction for the leaves of four types of winter wheat (Pdl, Per, Ods, Dos) (*red*); the results of approximation by three exponential components (*black*)

It was shown that there are only three components in the exponential decomposition of the kinetic curve of fluorescence induction for the leaves of plants of different genotypes grown in normal conditions, and the sum of these components well correlates with the experimental curve (Fig. 4).

Table 1 shows the results of decomposition of induction curves of chlorophyll fluorescence for four types of winter wheat.

**Table 1:** The results of decomposition of induction curves of chlorophyll fluorescence for four types of winter wheat

Type of winter wheat	Weighted contributions of exponential components *a*_1_, *a*_2_, *a*_3_ %	Rate constants *k*_1_, *k*_2_, *k*_3_c^−1^	Chl *a*/*b*	Ratio *a*_1/_ *a*_2/_ *a*_3_
Pdl	59, 29, 7	400, 87, 11	3.59	8/4/1
Per	67, 23, 3	300, 57, 5	3.52	22/8/1
Ods	67, 22, 4	300, 66, 12	3.91	17/6/1
Dos	77, 22, 4	370, 71, 13	3.60	19/6/1

The values of exponential components for four leaf genotypes are close to each other. The ratio of the weights of exponential components with decrements (*k*_*1*_ *> k*_*2*_ *> k*_*3*_) is approximately *a*_*1*_ *:a*_*2*_ *:a*_*3*_ = 10/5/1. Electron microscopic studies of thale cress (*Arabidópsis thaliána*) and spinach (*Spinácia olerácea)* [11] showed that the ratio of the detected three configurations of PSII pigment-protein complexes was 8:2:1, that is close to the above ratio *a*_*1*_ *:a*_*2*_ *:a*_*3*_ (see Table 1).

## Conclusions

A computer program for the decomposition of the basic reaction kinetics of biological macromolecules into exponential components has been developed. The program was tested for analysis of the kinetics of photoinduced charge transfer in the reaction centers (RCs) of purple bacteria and the induction of fluorescence in granum thylakoid membranes. An identification of the kinetics of structural changes in RC in the process of photoexcitation was carried out with this program. It was found that after photoexcitation of RC, its structure had the most non-equilibrium character within the first 40 s after switching off the light. The dark state was achieved by RC in the process of relaxation after turning off the light, and dark values of absorption were achieved after additional exposure of RC without light for 700 s.

Examination of fluorescence induction of PSII complexes in four genotypes of winter wheat of different ages showed that only three exponential components were present in the kinetics of induction, with the ratio of weights *a*_*1*_ *:a*_*2*_ *:a*_*3*_ = 10/5/1. This value is close to the ratio of 8:2:1 for three configurations of PSII pigment-protein complexes, obtained by electron microscopic studies of thale cress (*Arabidópsis thaliána*) and spinach (*Spinácia olerácea)*. This indicates on possible correlation between the exponential components of fluorescence induction of PSII complexes and the composition of pigment-protein complexes of granum thylakoid membranes. It provides an opportunity to replace expensive electron microscopic investigations by much easier studies of the kinetics of fluorescence induction in granum thylakoid membranes.
